# Long non-coding RNA *Tug1* modulates mitochondrial and myogenic responses to exercise in skeletal muscle

**DOI:** 10.1186/s12915-022-01366-4

**Published:** 2022-07-18

**Authors:** Adam J. Trewin, Jessica Silver, Hayley T. Dillon, Paul A. Della Gatta, Lewan Parker, Danielle S. Hiam, Yin Peng Lee, Mark Richardson, Glenn D. Wadley, Séverine Lamon

**Affiliations:** 1 Institute for Physical Activity and Nutrition, and School of Exercise and Nutrition Sciences, Deakin University, Geelong, Australia; 2 Human Integrated Physiology and Sports Cardiology Laboratory, Baker Heart and Diabetes Institute, Melbourne, Australia; 3 Institute for Health and Sport (iHeS), Victoria University, Melbourne, Australia; 4 Genomics Centre, School of Life and Environmental Sciences, Faculty of Science, Engineering and Built Environment, Deakin University, Waurn Ponds, Victoria Australia

**Keywords:** Muscle, Transcriptome, Non-coding RNA, Bioenergetics

## Abstract

**Background:**

Mitochondria have an essential role in regulating metabolism and integrate environmental and physiological signals to affect processes such as cellular bioenergetics and response to stress. In the metabolically active skeletal muscle, mitochondrial biogenesis is one important component contributing to a broad set of mitochondrial adaptations occurring in response to signals, which converge on the biogenesis transcriptional regulator peroxisome proliferator-activated receptor coactivator 1-alpha (PGC-1α), and is central to the beneficial effects of exercise in skeletal muscle. We investigated the role of long non-coding RNA (lncRNA) taurine-upregulated gene 1 (*TUG1*), which interacts with PGC-1α in regulating transcriptional responses to exercise in skeletal muscle.

**Results:**

In human skeletal muscle, *TUG1* gene expression was upregulated post-exercise and was also positively correlated with the increase in PGC-1α gene expression (*PPARGC1A*). *Tug1* knockdown (KD) in differentiating mouse myotubes led to decreased *Ppargc1a* gene expression, impaired mitochondrial respiration and morphology, and enhanced myosin heavy chain slow isoform protein expression. In response to a Ca^2+^-mediated stimulus, *Tug1* KD prevented an increase in *Ppargc1a* expression. RNA sequencing revealed that *Tug1* KD impacted mitochondrial Ca^2+^ transport genes and several downstream PGC-1α targets. Finally, *Tug1* KD modulated the expression of ~300 genes that were upregulated in response to an in vitro model of exercise in myotubes, including genes involved in regulating myogenesis.

**Conclusions:**

We found that *TUG1* is upregulated in human skeletal muscle after a single session of exercise, and mechanistically, *Tug1* regulates transcriptional networks associated with mitochondrial calcium handling, muscle differentiation and myogenesis. These data demonstrate that lncRNA *Tug1* exerts regulation over fundamental aspects of skeletal muscle biology and response to exercise stimuli.

**Supplementary Information:**

The online version contains supplementary material available at 10.1186/s12915-022-01366-4.

## Background

Mitochondria are an integral metabolic hub of fundamental biological processes including the maintenance of cellular bioenergetics, ion homeostasis and redox signalling [1, 2]. In metabolically active tissues, such as skeletal muscle, the mitochondrial network exists as a dynamic system that can respond to environmental and physiological signals and adapt to improve cellular stress resistance to a subsequent challenge [3, 4]. A major component of these adaptations is mitochondrial biogenesis, a process involving a coordinated increase in the synthesis of mitochondrial constituents [5]. Mitochondrial biogenesis is transcriptionally regulated by peroxisome proliferator-activated receptor gamma coactivator 1-alpha (PGC-1α), encoded by the gene *PPARGC1A*, which coordinates a program of gene expression from both the nuclear and mtDNA genomes [6].

Exercise is well-known to stimulate PGC-1α-mediated mitochondrial biogenesis [7]. Various exercise-induced signal transduction pathways converge on PGC-1α. These include 5′ adenosine monophosphate-activated protein kinase (AMPK), which senses the perturbation to cellular energy state; p38 mitogen-activated protein kinase (p38-MAPK), which is sensitive to cell stress signals; and Ca^2+^/calmodulin-dependent protein kinase (CaMK), which is sensitive to the intracellular Ca^2+^ fluxes involved in muscle contraction. These kinases and other upstream factors activate PGC-1α via post-translational modification (i.e. phosphorylation), which promotes interaction with various transcription factors to induce expression of target genes involved in mitochondrial biogenesis and cellular metabolism. These target genes notably include *PPARGC1A* itself, enabling autoregulatory positive feedback of its expression [5, 8]. Although mitochondrial adaptations are central to the overall health benefits that exercise elicits [5], we do not yet have a complete understanding of the factors that regulate PGC-1α in response to exercise.

There is increasing evidence demonstrating the importance of regulation by a class of transcripts known as long non-coding RNA (lncRNAs) [9]. lncRNAs are arbitrarily defined as being >200 *nt* in length and lack protein-coding potential [10]. lncRNAs can regulate transcriptional and translational processes via physical interactions with genes, transcripts and proteins [9] including those that constitute mitochondria [11]. The lncRNA taurine-upregulated gene 1 (*TUG1*) is well-conserved between rodents and humans [12] and was recently reported to positively regulate PGC-1α in murine kidney cells [13]. Their work suggests that *Tug1* functions by forming a scaffold between PGC-1α protein and a region of DNA upstream of the *Ppargc1a* promotor, which enhances *Ppargc1a* expression [13].

Given this, and the central role the PGC-1α pathway plays in mediating mitochondrial adaptations to exercise [7], we aimed to investigate the role of *Tug1* in regulating skeletal muscle mitochondria. We hypothesised that lncRNA *Tug1* would be required for achieving full activation of the PGC-1α-mediated transcriptional program in skeletal muscle in response to an exercise stimulus. Here we report that *TUG1* expression is increased in human skeletal muscle in response to a bout of acute exercise. We also show that *Tug1* knockdown in mouse myocytes had surprisingly nuanced effects on *Ppargc1a* expression and mitochondrial function, yet unexpectedly marked effects on a wide range of transcriptional responses to exercise-like stimuli, including calcium handling and myogenesis.

## Results

### Post-exercise *TUG1* lncRNA expression is increased in human skeletal muscle

**Fig. 1:**
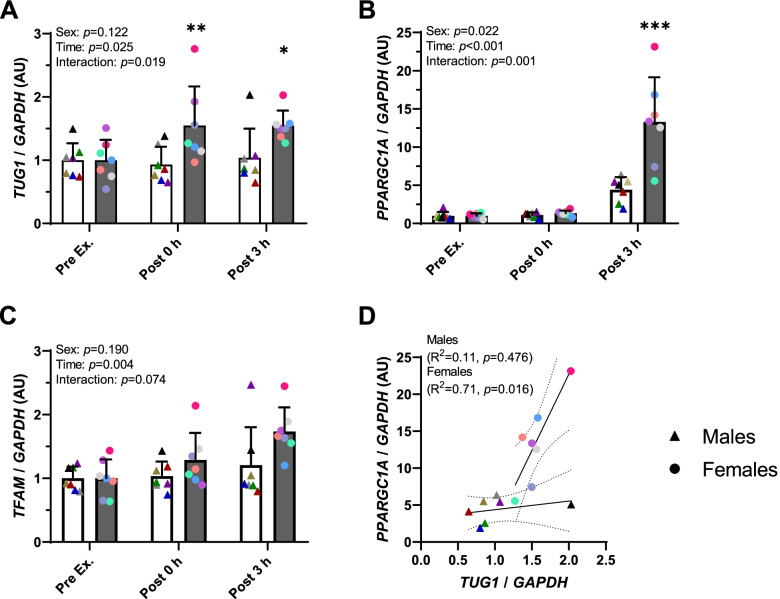
lncRNA *TUG1* expression is increased in human skeletal muscle in response to a single bout of aerobic exercise. Gene expression of **A ***TUG1*, **B ***PPARGC1A* and **C ***TFAM* measured by RT-qPCR from muscle (*vastus lateralis*) biopsy samples before (Pre Ex), immediately (Post 0 h) and 3 h after (Post 3 h) a 60-min bout of cycling exercise at 70% VO_2peak_. Matching point colours represent the same male (triangles, open bars) and female (circles, shaded bars) participants across all time points; bars are mean (SD) for *n*=7 subjects/sex. Data were analysed by repeated measures two-way ANOVA for main effects of sex and time. Significant interactions were analysed with Tukey post hoc test: *p < 0.05, **p < 0.01, ***p < 0.001 male vs. female. **D** Correlation between *TUG1* and *PPARGC1A* in males and females at Post 3 h; dotted lines represent 95% CI. See also Additional file 1: Supplementary Fig. S1

The lncRNA *Tug1* was recently shown to act as a positive regulator of PGC-1α in mouse kidney cells [13]. We therefore hypothesised that changes in *TUG1* would be associated with increases in *PPARGC1A* expression that occurs in human skeletal muscle following an acute bout of aerobic exercise [14, 15]. To investigate this, gene expression was measured in skeletal muscle from a cohort of young healthy males and females (*n* = 7 per sex) who performed moderate-intensity aerobic exercise for 1 h, with muscle biopsies obtained before, immediately after and 3 h after exercise. Before exercise, the expression of *TUG1* was not different between males and females at baseline (Fig. 1A and Additional file 1: Fig. S1). Immediately and 3 h after exercise, *TUG1* expression was increased by ~1.5-fold from baseline in females (*p < 0.05* for both time points), but not in males (Fig. 1A), despite both sexes performing exercise at the same relative intensity (70% VO_2peak_; Table 1). The increased expression of *PPARGC1A* 3 h after exercise (main effect of time, *p* < 0.001) was also more robust in females compared to males (*p* < 0.05 3 h after exercise, Fig. 1B). Mitochondrial transcription factor A (*TFAM*), a key downstream PGC-1α target gene, also increased with exercise (main effect of time, *p* < 0.01) with no statistically significant effect of sex (Fig. 1C). Intriguingly, there was a strong positive linear correlation between increases in *TUG1* and *PPARGC1A* expression levels at 3 h post-exercise in females (*R*^2^ = 0.72, *p = 0.02*), but not in males (*R*^2^ = 0.11, *p = 0.47*; Fig. 1D). Taken together, these data suggest that *TUG1* may be involved in the PGC-1α-mediated response to exercise in skeletal muscle, with a sex-specific effect.

**Table 1:** Participant characteristics

	Male	Female	***p*** value
Age (years)	23.6 ± 4.8	23.6 ± 3.3	1.000
Weight (kg)	74.0 ± 7.4	66.1 ± 8.3	0.085
BMI (kg/m^2^)	24.2 ± 3.1	24.5 ± 2.7	0.859
VO_2peak_ (L/min)	3.55 ± 0.37	2.63 ± 0.27	0.000
VO_2peak_ (mL/min/kg)	47.9 ± 6.3	40.1 ± 5.0	0.026
Power (W) at 70% VO_2peak_	146.6 ± 25.5	102.4 ± 14.0	0.002

Values are mean ± SD, *n* = 7 per sex; two-tailed unpaired *t*-test

### *Tug1* regulates mitochondria- and myogenesis-related gene and protein expression, in vitro

To understand the potential mechanistic relationship between *TUG1* and PGC-1α in the skeletal muscle response to exercise, we conducted in vitro studies using the C2C12 cell line, originally derived from skeletal muscle of a female C3H mouse [16–18]. Knockdown (KD) of *Tug1* (the *Mus musculus* orthologue of human *TUG1*) was achieved using antisense locked nucleic acid (LNA) oligonucleotides, which hybridise with their target sequence, resulting in cleavage by endogenous RNase-H activity [19]. The LNA was designed to target all three transcript variants of *Tug1* (Additional file 1: Fig. S2A). This was confirmed by qPCR (Additional file 1: Fig. S2B), and transfection of the LNA at 5–50 nM concentrations decreased levels of *Tug1* to approximately 40% of the negative control LNA (herein referred to as *control*) (Additional file 1: Fig. S2C).

**Fig. 2:**
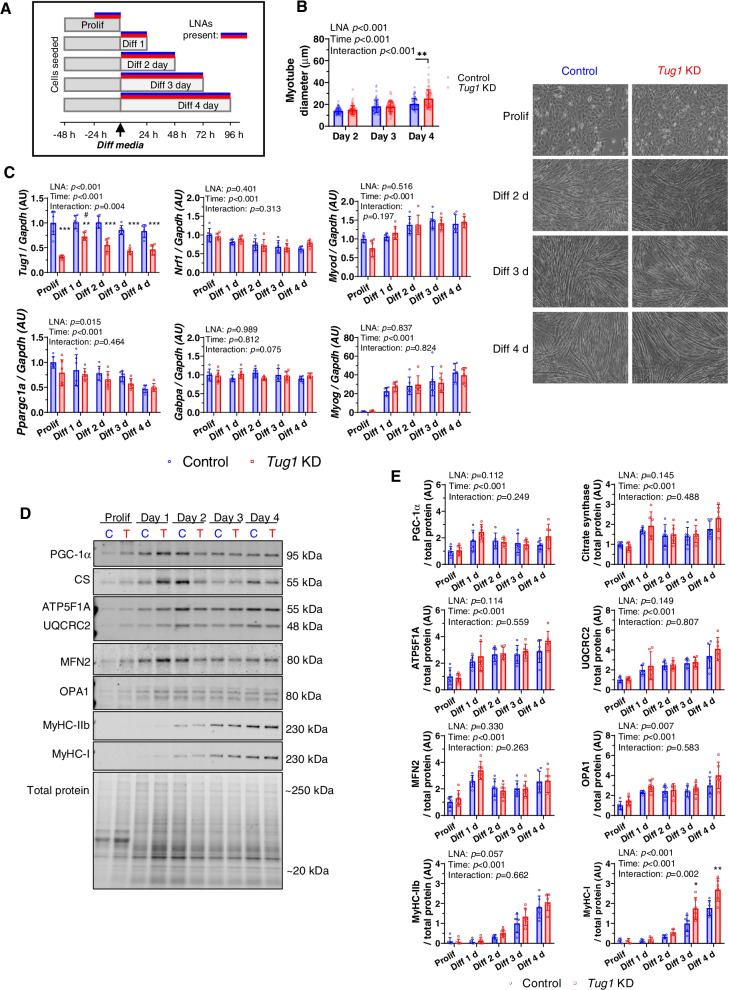
Mitochondria and myogenesis-related gene and protein levels in response to *Tug1* knockdown during myocyte differentiation, in vitro. **A** C2C12 myoblasts were transfected with *Tug1* or control LNAs (25 *n*M) for 24 h during proliferation (Prolif) or for 1 to 4 days during differentiation (Diff). **B** Quantification of myotube diameter and representative phase-contrast images. Data are measurements of ≥20 individual myotubes per well from *n*=3 replicate wells, analysed by two-way ANOVA for main effects of LNA and time, with significant interactions analysed by Bonferroni post hoc tests: **p < 0.01 *Tug1* KD compared to control. **C** Gene expression of selected targets by RT-qPCR, *n*=6 replicates per group and time point. **D** Representative immunoblots of protein expression in control (C) and *Tug1* KD (T) samples **E** quantified relative to total protein (stain free); data are mean (SD) for *n*=6 (triplicate wells across two independent experiments), analysed by two-way ANOVA for main effects of LNA and time, with significant interactions analysed by Bonferroni post hoc tests: *p < 0.05, **p < 0.01, ***p < 0.001 *Tug1* KD compared to control. See also Additional file 1: Supplementary Fig. S2

Using this model, we first sought to determine the effects of *Tug1* knockdown in myotube differentiation — a process that requires mitochondrial biogenesis [20, 21]. *Tug1* was knocked down for 24 h in proliferating myoblasts and for 24, 48, 72 and 96 h during differentiation into myotubes (Fig. 2A). Interestingly, *Tug1* KD appeared to augment myotube diameter by day 4 of differentiation (Fig. 2B). Analysis of gene expression by qPCR showed that there was a main effect of *Tug1* KD to decrease the expression levels of *Ppargc1a*; however, downstream targets *Nrf1* (nuclear respiratory factor 1) and *Gabpa* (GA-binding protein alpha) remained unaffected (Fig. 2C). The expression of transcription factors *Myod1* (myogenic differentiation 1) and *Myog* (myogenin) increased as expected throughout differentiation; however, their mRNA abundance was not significantly affected by *Tug1* KD (Fig. 2C).

At the protein level, knockdown of *Tug1* throughout differentiation did not appear to affect PGC-1α protein abundance (LNA main effect *p* = 0.11; Fig. 2D, E). Similarly, throughout differentiation, *Tug1* KD did not significantly alter levels of mitochondrial proteins including citrate synthase, ATP5F1A and UQCRC2 (subunits of complexes V and III, respectively), or mitofusin 2 (MFN2) — an outer mitochondrial membrane fusion protein transcriptionally regulated by PGC-1α [22] (Fig. 2D, E). There was however a significant main effect of *Tug1* KD to increase expression of OPA1, a GTPase involved in regulating inner mitochondrial membrane fusion (Fig. 2D, E). Lastly, as a marker of myotube differentiation, we measured myosin heavy chain (MyHC) protein expression. Notably, we found that *Tug1* KD augmented the increase in MyHC type I (slow) isoform by day 3 and 4 of differentiation, whereas the increase in MyHC type IIb (fast) isoform was unaffected (Fig. 2D, E).

### Tug1 regulates mitochondrial morphology and respiratory function, in vitro

**Fig. 3:**
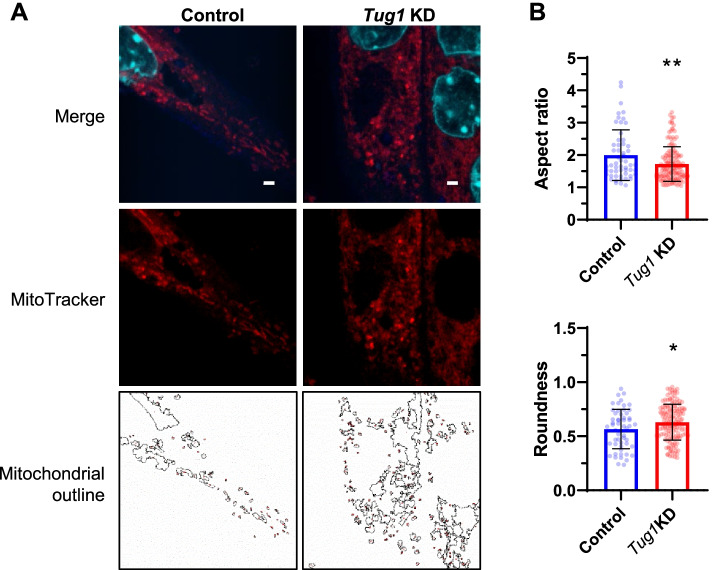
Mitochondrial morphology is more fragmented in *Tug1* KD myotubes. C2C12 myocytes were differentiated for 3 days, transfected with *Tug1* and control LNAs (25 *n*M) for 24 h. **A** Confocal microscopy of mitochondria (red channel; MitoTracker RedCMXros, 50 *n*M), F-actin (blue channel; phalloidin) and nuclei (cyan; DAPI). Scale bar = 2 μm. **B** Morphology analyses of mitochondria shown in **A***.* Individual points are the quantification of each outlined region and bars represent the mean (SD) for one replicate analysed by unpaired two-tailed *t*-test: *p<0.05, **p<0.01. See also Additional file 1: Supplementary Fig. S3; individual data are available at DOI: 10.6084/m9.figshare.20175770 [24]

**Fig. 4:**
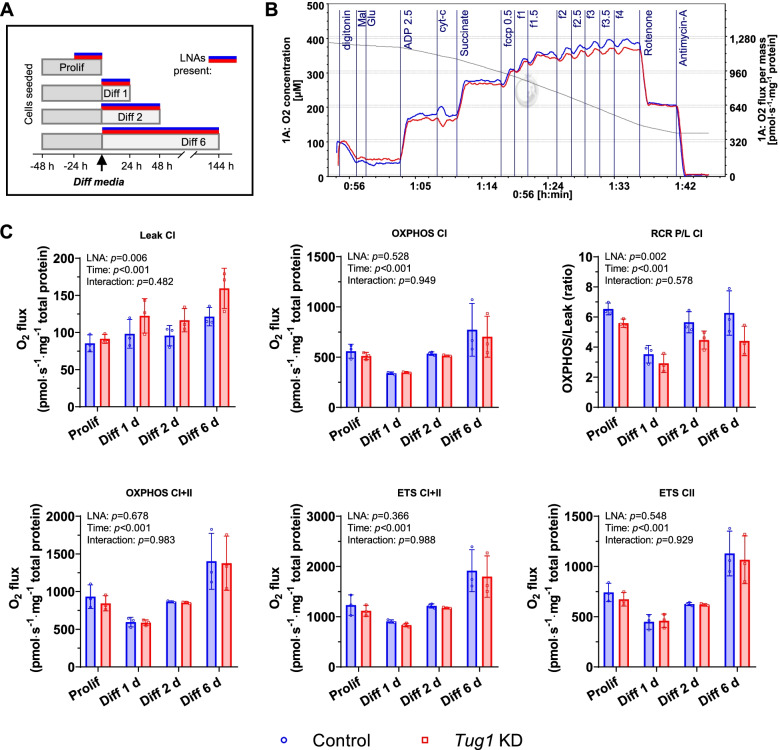
Mitochondrial leak state respiration is elevated in *Tug1* KD myoblasts and differentiating myotubes. **A** C2C12 cells were transfected with antisense LNAs (10 *n*M) for *Tug1* knockdown (*Tug1* KD) or control then proliferated (Prolif) for 24 h or differentiated (Diff) for 1, 2 or 6 days prior to high-resolution mitochondrial respiratory analyses. **B** Representative trace showing O_2_ flux for control (blue) overlaid with *Tug1* KD (red) throughout a substrate, uncoupler and inhibitor titration (SUIT) protocol. **C** Quantification of O_2_ consumption during specific respiratory states (Leak, non-phosporylating O_2_ flux; OXPHOS, ADP stimulated oxidative phosphorylation; ETS, uncoupled electron transport system) supported by complex I linked (malate + glutamate) and complex II linked (succinate) substrates, normalised to total cellular protein. Data are mean (SD) for *n*=3 independent experiments for each time point, analysed by ordinary two-way ANOVA for main effects of LNA and time, with Bonferroni post hoc tests for significant interaction effects: *p<0.05 *Tug1* KD compared to NC. See also Additional file 1: Supplementary Fig. S4; individual data are available at DOI: 10.6084/m9.figshare.20175770 [24]

Despite the limited effects of *Tug1* KD on the expression of several mitochondrial genes and proteins measured throughout myocyte differentiation, it is possible that additional factors could affect mitochondrial morphology and respiratory function. We found that mitochondria in *Tug1* KD myotubes were less elongated (as indicated by aspect ratio measurements) and more rounded (Fig. 3 and Additional file 1: Fig. S3), suggesting a shift towards a more fragmented morphology. Fragmented mitochondria are known to have elevated rates of leak state respiration which impairs electron transport system coupling efficiency [3, 23]. Therefore, we next measured the effects of *Tug1* KD on mitochondrial respiration (O_2_ consumption) in myoblasts and differentiated myotubes using high-resolution respirometry (Fig. 4A, B, and Additional file 1: Fig. S4). Rates of leak state (non-phosphorylating) respiration supported by complex I substrates (LEAK CI) were elevated with *Tug1* KD compared to control myotubes (Fig. 4C). Rates of oxidative phosphorylation supported by complex I substrates (OXPHOS CI) were not significantly affected by *Tug1* KD; however, there was a significant impairment in respiratory coupling ratio (RCR P/L CI, Fig. 4C). Uncoupled respiration rates supported by convergent complex I+II substrate input (ETS CI+II) or complex II alone (ETS CII) were not significantly affected by *Tug1* KD (Fig. 4C).

### *Tug1* regulates *Ppargc1a* mRNA responses to pharmacological exercise-mimetics in vitro

**Fig. 5:**
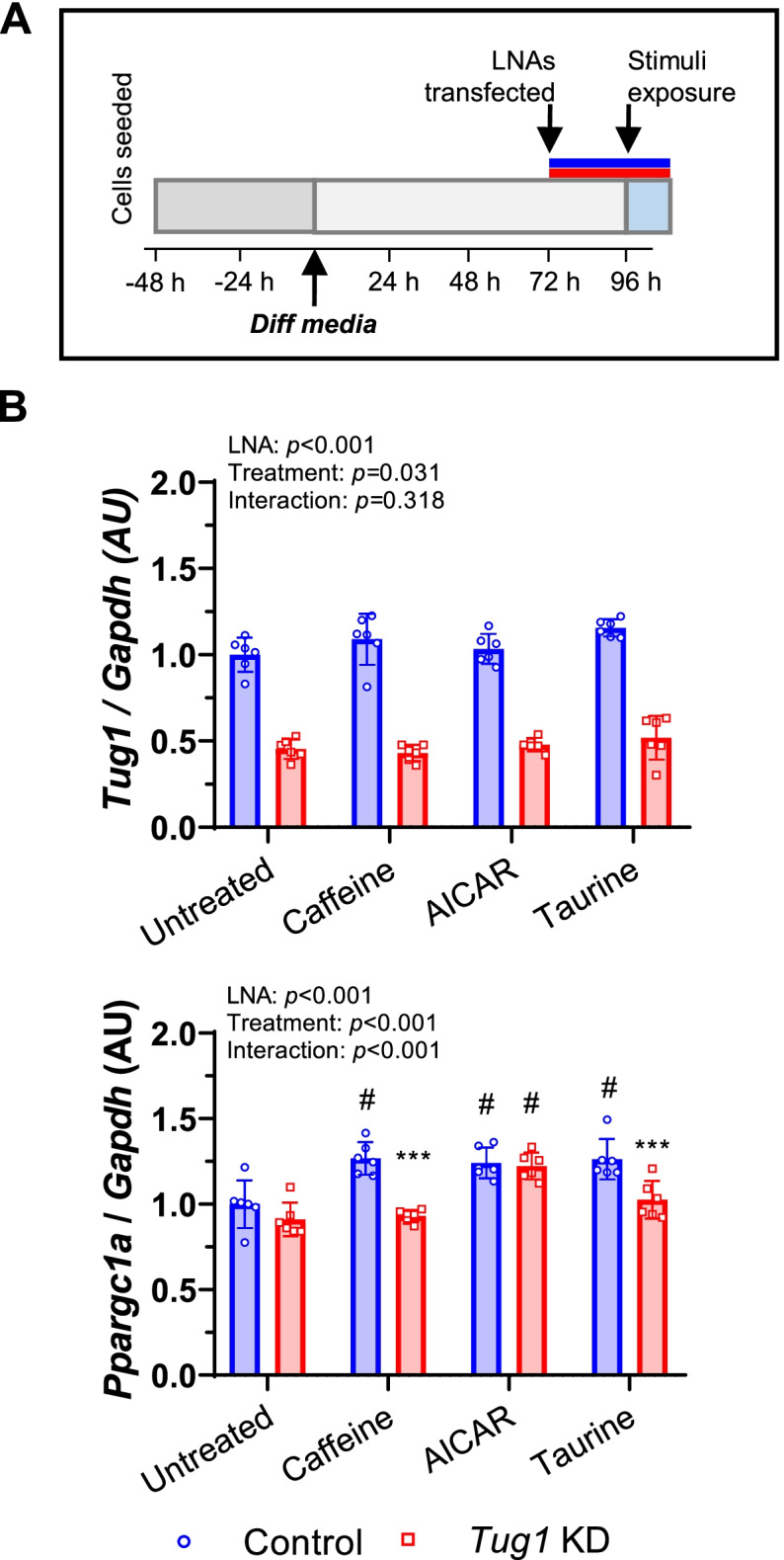
*Ppargc1a* expression in response to a Ca^2+^-mediated pharmacological exercise mimetic requires *Tug1*. **A** C2C12 myocytes were differentiated for 3 days then transfected with *Tug1* or control LNA (25 *n*M) for 24 h. Transfected myotubes were then exposed to caffeine (0.1 mM), AICAR (0.5 mM), taurine (5 mM) or untreated control for 6 h, then **B** gene expression of *Tug1* and *Ppargc1a* was analysed by RT-qPCR. Data are mean (SD) for *n*=6 (triplicate wells across two independent experiments), analysed by ordinary two-way ANOVA for main effects of LNA and treatment. Significant main interaction effect was analysed by Bonferroni post hoc test; **p<0.01, ***p<0.001, *Tug1* KD vs. control (NC)

We next sought to understand how *Tug1* KD affects *Ppargc1a* mRNA expression in response to cellular signals associated with exercise. To do this, we pharmacologically mimicked two key exercise-mediated signals known to stimulate PGC-1α-mediated transcriptional activity then measured subsequent changes in the expression of *Ppargc1a* mRNA (Fig. 5A). Elevations in sarcoplasmic [Ca^2+^] that occur in muscle contraction were mimicked by incubating myotubes with caffeine to stimulate Ca^2+^ release from the sarcoplasmic reticulum [25]. Perturbations to cellular energy homeostasis that occur during exercise were mimicked using the AMPK agonist, 5-aminoimidazole-4-carboxamide ribonucleotide (AICAR) [26]. There was a main effect of treatment on *Tug1* gene expression, which tended to increase in response to taurine (used here as a positive control, i.e. *t*aurine-*u*pregulated *g*ene) (Fig. 5B). In control cells, *Ppargc1a* mRNA expression increased as expected in response to caffeine, AICAR and taurine. However, *Tug1* KD prevented the increase in *Ppargc1a* mRNA expression in response to caffeine and taurine, but not AICAR (Fig. 5B). This suggests that *Tug1* plays a role in Ca^2+^-mediated but not AMPK-mediated expression of *Ppargc1a* mRNA.

### *Tug1* regulates muscle transcriptomic responses to exercise-like stimuli, in vitro

**Fig. 6:**
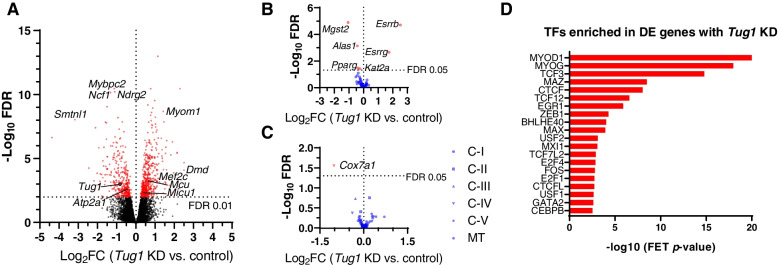
Transcriptomic responses to *Tug1* KD in myotubes. C2C12 myocytes were differentiated for 3 days, transfected with *Tug1* or control LNAs for 24 h, then harvested on day 4 for RNA-seq. **A** Volcano plot of 867 differentially expressed genes (FDR<0.01, red points) that were upregulated (positive log_2_FC) or downregulated (negative log_2_FC) as a result of *Tug1* KD compared to control. Selected genes of interest are labelled (see supplementary tables for full list). **B** Volcano plot of PGC-1α target genes with *Tug1* KD compared to control. **C** Volcano plot of nuclear-encoded mitochondrial ETC subunits of complexes *I* to *V* (C-I – C-V) as well as mtDNA-encoded (MT) subunit expression with *Tug1* KD compared to control. **D** Transcription factors enriched among DE genes due to *Tug1* KD. Data are from *n*=6 libraries per group (triplicate wells from two independent experiments for each condition). See also Additional file 1: Supplementary Fig. S5, as well as Additional file 2: Supplementary tables S1 and S2

To further understand the role of *Tug1* on the muscle transcriptome, we performed RNA sequencing on myotubes at rest and in response to an exercise-like stimulus. At rest, *Tug1* KD was associated with the downregulation of 351 genes and upregulation of 516 genes (FDR < 0.01; Fig. 6A, Additional file 1: Figure S5 and Additional file 2: Table S1). The most highly enriched biological process based on gene ontology (GO) overrepresentation analysis (Additional file 2: Table S2) was “positive regulation of mitochondrial calcium ion concentration (GO:0051561)” which was associated with the upregulation of *Mcu* (mitochondrial calcium uniporter), *Micu1* and *Micu2* (mitochondrial calcium uptake) and *Mcur1* (mitochondrial calcium uniporter regulator 1), yet downregulation of *Atp2a1* (sarcoplasmic/endoplasmic reticulum calcium ATPase 1). We confirmed that *Mcu* upregulation was a *Tug1*-specific effect by observing the same response with an alternative *Tug1* LNA in a different cell line (Additional file 1: Fig. S5G). Also enriched in response to *Tug1* KD were terms including “regulation of striated muscle contraction (GO:0006942)” and “muscle cell development (GO:0055001)” (Additional file 2: Table S2). Evaluation of 80 PGC-1α target genes (defined as containing one or more binding sites for PGC-1α in their promoter region) showed that 4 were downregulated (*Pparg*, *Alas1*, *Kat2a* and *Mgst2*) and 2 upregulated (*Esrrb* and *Esrrg*) in response to *Tug1* KD (FDR < 0.05; Fig. 6B). However, downstream PGC-1α target genes directly involved in mitochondrial biogenesis such as *Nrf1*, *Gabpa* and *Tfam* were unaffected. In line with this, the expression of genes encoding mitochondrial electron transport chain subunits were unaffected by *Tug1* KD, with the exception of a muscle-specific complex IV subunit (*Cox7a1*) which was downregulated (Fig. 6C). Transcription factor enrichment analysis revealed that the myogenic transcription factors MYOD1 and MYOG may be involved in mediating the effects of *Tug1* KD on the myotube transcriptome (Fig. 6D).

**Fig. 7:**
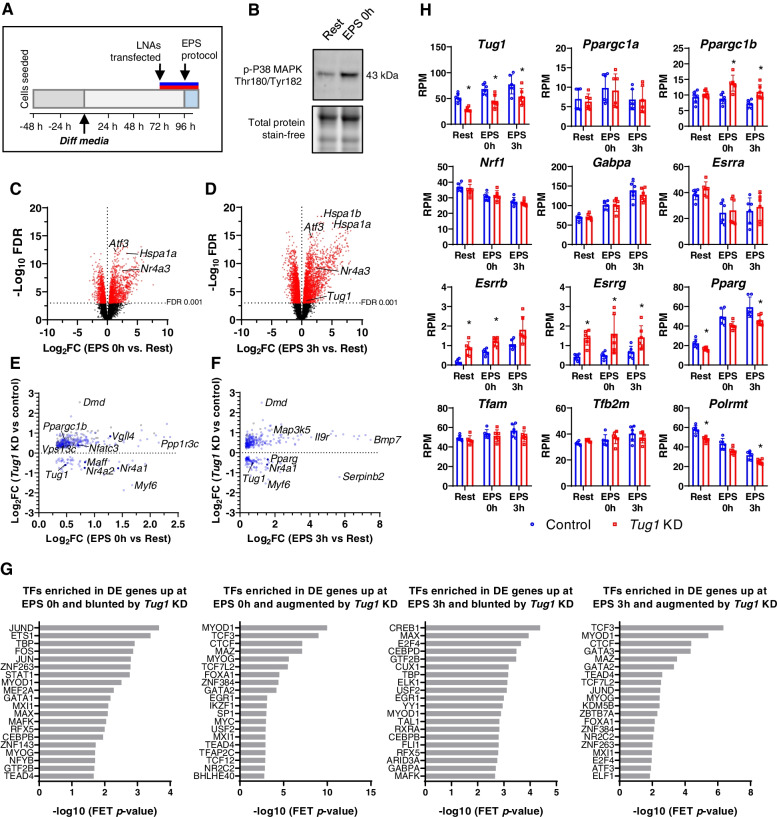
*Tug1* KD modifies transcriptomic responses to simulated exercise in myotubes. **A** C2C12 myocytes were differentiated for 3 days, transfected with *Tug1* or control LNAs for 24 h, then on day 4 underwent electrical pulse stimulation (EPS) or rest for 3 h before being harvested immediately (EPS 0h) or after 3-h recovery (EPS 3h) for RNA-seq. **B** Representative immunoblot of phosphorylated p38 MAPK immediately after 3 h EPS. **C** RNA-seq volcano plot of genes in control myotubes differentially expressed immediately after the EPS protocol, and **D** 3 h after EPS compared to Rest (red points, FDR<0.001). **E** Scatterplot of genes that were upregulated (FDR<0.05) at EPS 0h or **F** EPS 3h compared to Rest, but blunted or augmented by *Tug1* KD compared to control (FDR<0.05); grey points represent genes upregulated only in *Tug1* KD myotubes after EPS but that were not upregulated in control myotubes after EPS. **H** RNA-seq data in reads per million (RPM) for *Tug1* and selected genes associated with mitochondrial biogenesis. **G** Transcription factors enriched in genes (shown in panels **E** and **F**) that were upregulated by EPS yet blunted or augmented due to *Tug1* KD. FET, Fisher’s exact test. Data are from *n*=6 libraries per group (triplicate wells from two independent experiments for each condition); *FDR<0.05 *Tug1* KD vs. NC. See also Additional file 1: Supplementary Fig. S5, as well as Additional file 2: Supplementary Tables S3 to S9

To simulate exercise in vitro, we used electrical pulse stimulation (EPS) to induce myotube contractions [27]. Myotubes were transfected with LNAs at day 3 of differentiation; then, on day 4, they were electrically stimulated for 3 h. Cells were harvested immediately after EPS (EPS 0h), or after 3 h of recovery (EPS 3h), then analysed by RNA sequencing (Fig. 7A). At EPS 0 h, there was robust EPS-induced phosphorylation of P38 MAPK (Fig. 7B), which is a typical response to acute exercise [28] and there were 835 genes upregulated compared to baseline (Rest) in control myotubes (log_2_FC>1, FDR<0.01; Fig. 7C, Table S3). Among these were *Hspa1a*, *Atf3* and *Nr4a3* which are well-established exercise-responsive genes in human skeletal muscle [14, 29]. Following 3-h recovery (EPS 3h), 1316 genes were upregulated compared to Rest in control myotubes (log_2_FC>1, FDR<0.01; Fig. 7D, Additional file 2: Table S4), 764 of which were common to those at EPS 0 h while 551 were unique to EPS 3 h (Additional file 1: Fig. S5C). There was a significant overlap of 44 genes downregulated by *Tug1* KD and also downregulated 3 h after EPS (Additional file 1: Fig. S5D).

Of particular interest were 53 genes at EPS 0 h and 101 genes at EPS 3 h whose upregulation in response to EPS was blunted by *Tug1* KD (Fig. 7E, F, and Additional file 2: Tables S5 and S6). Among these were *Nr4a1* (nuclear receptor subfamily 4, group A, member 1), which was recently identified as an important myogenic factor in muscle [30], and *Maff* (MAF bZIP transcription factor F) and *Myf6* (myogenic factor 6), which are both involved in skeletal muscle differentiation, in addition to *Tug1* itself. Several GO biological process terms were associated with these genes that relate to regulation of transcription and metabolic processes (Additional file 2: Table S7). There were also 338 genes at EPS 0 h and 254 genes at EPS 3 h whose upregulation in response to EPS was augmented by *Tug1* KD (Fig. 7E, F, and Additional file 2: Tables S8 and S9). Notable genes included *Dmd*, which encodes dystrophin — a structural component of the sarcolemma; *Ppp1r3c*, involved in regulating glycogen synthase activity; *Vps13c*, involved in maintenance of mitochondrial membrane potential; and *Vgll4*, which was recently shown to play a role in muscle differentiation [31]. Transcription factor enrichment analysis on these DE genes suggests that *Tug1* regulates myogenic signalling in response to EPS, with the transcription factors MYOD1 and MYOG among the highest ranked (Fig. 7G).

*Ppargc1a* was not differentially expressed in response to EPS and/or *Tug1* KD at the FDR 0.05 level, potentially due to between-sample variability (Fig. 7H). We sought to clarify this result by also performing RT-qPCR on these samples, which showed that *Tug1* KD decreased *Ppargc1a* expression in these samples (Addition file 1: Fig. S5E). Nevertheless, RNA-seq showed that direct downstream mitochondrial biogenesis targets of PGC-1α such as *Nrf1* and *Tfam* were not upregulated by EPS other than *Gabpa*, and none was affected by *Tug1* KD (Fig. 7H). Further downstream, however, the mitochondrial RNA polymerase, *Polrmt*, was downregulated with *Tug1* KD at EPS 3 h (Fig. 7H). Similarly, *Esrrb* and *Esrrg* were downregulated, while *Pparg* was upregulated by *Tug1* KD after EPS. Interestingly, *Tug1* KD altered the expression of *Ppargc1b* in response to EPS: it was upregulated following EPS in *Tug1* KD myotubes, but not in control myotubes (Fig. 7H). Taken together, this demonstrates an unexpectedly complex role for *Tug1* in regulating a broad range of transcriptomic responses to exercise-like stimuli in skeletal muscle.

## Discussion

Here, we report for the first time the role of the lncRNA *TUG1* in skeletal muscle. We show that *TUG1* expression is increased in concert with *PPARGC1A* in the hours post-exercise in human female skeletal muscle. Using an in vitro *Tug1* knockdown approach in mouse myotubes, we demonstrate that *Tug1* has only subtle effects on the mitochondrial phenotype during myotube differentiation, but that *Tug1* regulates the expression of *Ppargc1a* in a Ca^2+^-dependent manner. Our major finding was that *Tug1* regulates the muscle transcriptome in response to an in vitro model of exercise, impacting upon a range of mitochondrial- and myogenesis-related gene networks.

Our analysis of human skeletal muscle revealed that females display an increase in *TUG1* expression in response to an acute bout of moderate-intensity aerobic exercise, yet males do not. This is intriguing, given that there was no difference in *TUG1* expression between males and females at baseline, which is consistent with skeletal muscle *TUG1* expression data reported elsewhere (Genotype-Tissue Expression Project, release version 8). It remains to be determined if this female post-exercise *TUG1* expression represents a bona fide sex-specific response, or whether similar responses would be found in males if the exercise stimulus was greater to achieve a similar *PPARGC1A* response to that of females. Nevertheless, the correlation between *TUG1* and *PPARGC1A* expression 3 h after exercise suggests a role for *TUG1* in the response to exercise in muscle.

We found that *Tug1* KD led to slight decreases in *Ppargc1a* gene expression in myotubes, but did not impair the expression of PGC-1α protein or downstream target genes that underpin the core mitochondrial biogenesis response, including *Nrf1*, *Gabpa* and *Esrra* or their respective target genes. Further downstream, *Polrmt* (mitochondrial RNA polymerase) was decreased with *Tug1* KD, suggesting that aspects of mtDNA transcription may have been impacted. However, initiation of mtDNA transcription requires POLRMT to form a complex with TFAM and TFB2M [32]. Neither of the latter were affected at the gene level by *Tug1* KD, and the expression of the resulting downstream mtDNA-encoded genes were also unchanged. Collectively, regulation of PGC-1α by *Tug1* appears relatively subtle under baseline conditions. This may be a muscle-specific effect, since previous findings in kidney cells showed that *Tug1* had a robust overall effect on mitochondrial biogenesis by directly regulating PGC-1α and its downstream target genes [13]. Another recent study found that overexpression of a putative open reading frame within *Tug1* in 3T3 and HeLa cells led to expression of a protein that could localise to mitochondria and regulate its membrane potential [33]. Given that this was an overexpression construct in vitro, it is unclear whether endogenous expression levels would be sufficient to regulate mitochondria, although it will be interesting to determine whether this occurs in muscle.

We found evidence that *Tug1* KD impacted several PGC-1α targets that are peripheral to the core mitochondrial biogenesis pathway. For example, the upregulation of *Pparg* in response to EPS was blunted by *Tug1* KD, which could potentially impact lipid metabolism [34] or have other deleterious effects, since knockout of *Pparg* in muscle elicits insulin resistance in vivo [35]. Similarly, genes encoding the oestrogen-related receptor family (ERRα/β/γ) displayed altered responses to *Tug1* KD. These nuclear receptors are targets of PGC-1α and regulate a network of genes involved in energy homeostasis. Although *Esrra*, which is well-known for its role in mitochondrial biogenesis, was unaffected, the responses of *Esrrb* and *Esrrg* to EPS were augmented by *Tug1* KD. Overexpression of *Esrrg* in mice causes a shift towards slow oxidative phenotype in muscle [36] and knockout impairs myogenesis [37]. To date, little is known about *Esrrb* in the context of muscle and exercise, though it is predicted that it could regulate a similar array of mitochondrial gene targets as the well-characterised *Esrra* [38].

Knockout studies in mice demonstrate that PGC-1α is sufficient, but not necessary for mitochondrial adaptations to exercise [39, 40]. Indeed, additional factors can regulate mitochondrial biogenesis in muscle [41] — a notion that our data supports. For instance, we found that *Ppargc1b* expression was unaffected by EPS in control myotubes, which is consistent with previous reports that it does not respond to exercise [42]; yet, we observed that it was upregulated in response to EPS in *Tug1* KD myotubes. Given that overexpression of PGC-1β can induce mitochondrial biogenesis [43, 44], our findings of an augmented *Ppargc1b* response to EPS with *Tug1* KD may represent a compensatory mechanism to maintain transcriptional activity of mitochondrial biogenesis when *Ppargc1a* expression is impaired.

Confocal microscopy revealed evidence of a shift towards a more fragmented mitochondrial morphology. Protein abundance of MFN2, the GTPase required for fusion events of the outer mitochondrial membrane and whose transcription is regulated by PGC-1α [22], was not impacted by *Tug1* KD in differentiating myotubes. However, expression of the inner mitochondrial membrane fusion protein, OPA1, was elevated. This potentially represents a compensatory response in an attempt to maintain an appropriate mitochondrial morphology. Regardless, the consequences of mitochondrial fragmentation could include impaired mitochondrial respiratory function [45], whereas fusion of the mitochondrial network allows tighter coupling of respiratory control to increase oxidative phosphorylation efficiency [46]. Indeed, our mitochondrial respiration data in which *Tug1* KD led to increased leak state respiration are consistent with this concept. Altogether, our data suggest that *Tug1* regulates (either directly or indirectly) mitochondrial morphology and respiratory coupling control.

In skeletal muscle in vitro, caffeine acts on ryanodine receptors to stimulate release of Ca^2+^ from the sarcoplasmic reticulum, mimicking excitation-contraction coupling [47]. We found that *Tug1* was required for the normal increase in *Ppargc1a* mRNA expression in response to caffeine. We speculate that in response to Ca^2+^-mediated activation of the CaMK pathway, *Tug1* may function to stabilise an interaction between transcription factors (such as CREB or MEF2) and PGC-1α, thereby regulating transcriptional activity at the *Ppargc1a* promotor. Indeed, both CREB and MEF2 were found to be among the most highly enriched transcription factors in response to EPS with *Tug1* KD (Fig. 7H). Further investigation is warranted to determine the precise mechanism involved. *Tug1* KD also led to altered expression of genes related to Ca^2+^ handling. This included downregulation of *Atp2a1*, which encodes the sarcoplasmic/endoplasmic reticulum calcium ATPase 1 (SERCA1), the major pump responsible for translocating Ca^2+^ from the cytosol to the SR for muscle relaxation. There was also upregulation of genes relating to the mitochondrial calcium uniporter (MCU) complex. This suggests that Ca^2+^ handling may have been impaired in *Tug1* KD myocytes and that MCU gene expression was upregulated to enhance mitochondrial Ca^2+^ uptake as a compensatory mechanism to maintain intracellular Ca^2+^ homeostasis. Taken together, this could have complex consequences for mitochondrial function [48].

We identified several exercise (EPS) and *Tug1*-responsive genes that are involved in the regulation of myogenesis. For instance, *Vgll4* (whose upregulation after EPS was augmented by *Tug1* KD) was recently shown to positively regulate myocyte differentiation through enhancing MYOG transactivation [31]. Additionally, we report upregulation of nuclear hormone receptor 4A (NR4A) genes *Nr4a1* and *Nr4a2* in response to EPS which were blunted by *Tug1* KD. This is pertinent for two reasons: firstly, *NR4A1*, in addition to isoforms 2 and 3, are among the most highly upregulated genes after acute aerobic exercise in humans [14], and secondly, *Nr4a1* is an important myogenic factor whose overexpression increases myosin slow isoform expression [30]. This could, in part, therefore explain the greater MyHC slow isoform protein expression that we observed in *Tug1* KD during differentiation. Another possible explanation for greater MyHC slow isoform protein expression is via an effect of *Tug1* on the calcineurin pathway. Calcineurin regulates muscle fibre-type via the combined activation of both MEF2 and NFAT transcription factors, which lead to expression of a slow-oxidative muscle fibre type gene program [49]. In the present study, *Tug1* KD led to upregulation of both of these genes (*Mef2c* and *Nfatc3*), suggesting a potential mechanism of fibre-type regulation by *Tug1*.

Non-coding RNAs in general, and particularly lncRNAs, can exert robust regulatory control over transcription and translation processes [9, 50, 51]. Given this, the marked impact of *Tug1* knockdown on the global muscle transcriptome is not surprising. The precise exercise-induced signals that upregulate *Tug1* expression remain to be elucidated. Potential candidates include a transcription factor (ChREBP) identified in a recent study that bound the *Tug1* promotor region under high-glucose conditions and repressed *Tug1* expression [52]. Additional transcription factor binding sites were also predicted in silico [52], including P53, which is known to be involved in mitochondrial responses to exercise [53]. It will now be crucial to experimentally determine the factors that regulate *Tug1* expression in response to exercise and how *Tug1* mechanistically interacts with putative targets besides PGC-1α.

## Conclusions

In summary, we report that *Tug1* regulates transcriptional responses to exercise-like stimuli by exerting nuanced regulatory effects on PGC-1α-related mitochondrial pathways, but also via modulation of calcium handling and myogenic signalling pathways in skeletal muscle. Muscle tissue is a major endocrine organ and determinant of overall metabolic health, and further understanding of the molecular regulators of mitochondria in skeletal muscle is imperative. Accordingly, lncRNA *Tug1* may be an attractive target for the development of new therapeutic strategies to treat numerous chronic diseases linked to metabolic impairments in skeletal muscle such as type 2 diabetes mellitus, obesity and cardiovascular diseases.

## Methods

### Human exercise study

Skeletal muscle biopsies were obtained as part of a study from our laboratory described elsewhere [54]. Briefly, the study was approved by Deakin University Human Research Ethics Committee (DUHREC 2014-096) and conformed to the Declaration of Helsinki. Written, informed consent was obtained from all participants before commencing exercise trials and sampling procedures. Female participants were taking oral contraceptives and performed the exercise protocol during the late follicular phase of the menstrual cycle. All participants initially performed a graded maximal exercise test on an electronically braked cycle ergometer (Lode, Groningen, the Netherlands) with breath-by-breath expired gas analysis (Innocor, Innovision, Glammsbjerg, Denmark) to determine peak oxygen uptake (VO_2peak_). On a separate occasion (at least 48 h later), participants cycled for 60 min at a workload corresponding to 70% VO_2peak_. Under local anaesthesia (1% Xylocaine), muscle biopsies were obtained from the *Vastus lateralis* of the same leg from separate incisions approximately 20 mm apart using a Bergstrom needle with suction at three time points: before, immediately post and 3 h post-exercise. Samples were snap frozen in liquid nitrogen and stored at −80°C until analysis. A subset of samples (*n*=7 male, *n*=7 female) from the original cohort were included in the present study based on available sample material. Participant anthropometrics are summarised in Table 1.

### Cell culture

C2C12 mouse myoblast cells were obtained from a commercial vendor (ATCC Cat# CRL-1772, RRID: CVCL_0188). Cells were cultured at 37°C with 5% CO_2_ in a humidified incubator in media consisting of Dulbecco’s modified Eagle medium (DMEM; Gibco #11995-065) supplemented with 1% v/v penicillin-streptomycin (Gibco #15140-122) and either 10% v/v FBS (Gibco #A3161001) for proliferation media or 2% v/v horse serum (Gibco #16050-130) for differentiation media. Cells were grown in T75 flasks to ~80% confluence then washed with sterile PBS wash and detached (Gibco TrypLE Express #LTS12604021) for passaging. Cells used for all experiments were ≤9 passages from stock and were seeded into 6-well plates for experiments unless otherwise stated.

### Gene silencing in vitro

Antisense locked nucleic acid (LNA) GapmeRs (Qiagen) were designed to target *mus musculus* lncRNA *Tug1* transcript variant *a* (NR_002321.2). The sequence was 5′-GAAGTTAAGCGTGAGA-3′ (Qiagen #339511 LG00228316-DDC) which also targets *Tug1* variants *b* and *c*. All knockdown experiments were performed in parallel with a negative control LNA: 5′-GCTCCCTTCAATCCAA-3′ (Qiagen #339515 LG00000001-DDC). Lyophilised LNAs were first resuspended in nuclease-free water to make a stock solution (50 μM). Lipofectamine® 2000 transfection reagent (Thermo Fisher Scientific) was diluted in serum-free DMEM (7% v/v) and in a separate tube, and LNAs were diluted in serum-free DMEM. The diluted LNAs and diluted Lipofectamine were then mixed 1:1 and incubated at room temperature for 15 min to form a final transfection mix. To transfect cells, 250 μL of transfection mix was then added per well of a 6-well plate of cells that already contained 1.75 mL of fresh proliferation or differentiation media, yielding a final LNA concentration per well of 25 nM unless otherwise stated. LNA transfection was performed for 24 h prior to subsequent experimental measurement unless otherwise stated.

### Myotube diameter

Phase-contrast images were obtained (TS100, Olympus) daily throughout differentiation. Myotube diameter was measured at the widest point using image software (ImageJ v1.51, NIH, USA). At least 20 myotubes were measured per image from 3 independent wells.

### In vitro exercise-like protocols

C2C12 myocytes were seeded in 6-well plates then grown to 90% confluence before switching to differentiation media. Differentiation media was replaced after 2 days; then, on day 3, LNA transfection was performed. Experiments were then conducted 24 h later on day 4 differentiated myotubes, as follows.

For pharmacological exercise mimetic experiments, myotubes were incubated with 0.5 mM 5-Aminoimidazole-4-carboxamide ribonucleotide (AICAR; A9978 Sigma Aldrich), 0.1 mM caffeine (C0750 Sigma Aldrich) or 5 mM taurine (T0625 Sigma Aldrich) and incubated for 6 h in a 37°C humidified incubator before media were removed and cells rapidly harvested in TRI-reagent as described below.

For electrical pulse stimulation (EPS) experiments, a commercially available system was used (C-PACE, IonOptix, MA, USA). Differentiation media volume was increased to 3 mL per well and pre-sterilised carbon electrodes were placed into the 6-well plates which were then returned to the 37°C humidified incubator. EPS was then performed for 3 h. Stimulation parameters were reproduced from a previous report [27] as follows: 13 V, 66 Hz, 2 ms pulse duration in trains of 5 s on and 5 s off. In parallel, plates of cells for the “Rest” condition had the carbon electrodes submerged into the media for the same duration but were not connected to the pulse generator. Immediately after the EPS protocol (or after 3-h recovery), the media were removed and cells were harvested in TRI-reagent as described below.

### RNA isolation and quality control

RNA was extracted from 5 to 12 mg frozen human skeletal muscle tissue in TRI-reagent (Ambion 15599018) using ceramic beads (Precellys CK28-R) with mechanical disruption at 6500 rpm for 30 s (MagnaLyser, Roche Diagnostics). Lysates were then frozen at −80°C until RNA isolation.

For RNA extraction from C2C12 cells, media were aspirated from each well at the end of an experiment, then cells were immediately lysed in 900 μL TRI-reagent (Ambion 15599018) with homogenisation achieved by pipette mixing. Lysates were then frozen at −80°C until RNA isolation as follows.

TRI-reagent lysates were thawed on ice and centrifuged at 10,000 × g for 10 min at 4°C. An aliquot of the supernatant was transferred to a new tube and mixed with an equal volume of 96% ethanol, then transferred to an RNA purification column with DNase-I treatment, as per manufacturer’s directions (Direct-Zol RNA Miniprep #R2052, Zymo Research). RNA concentration and purity (260:280 nm: >2.0) was determined with a spectrophotometer (NanoDrop 1000, Thermo Fisher Scientific).

### Reverse transcription and quantitative PCR (RT-qPCR)

Total RNA (1 μg) was reverse transcribed to first-strand cDNA in a 20-μL reaction along with no-template and no-RTase controls (Applied Biosystems High Capacity RT kit #4368814). Quantitative PCR was performed in triplicate (Agilent AiraMX G8830A) on 4 ng of cDNA (along with a no-template and a no-RT control) in a 10-μL reaction consisting of SYBR green master mix (Applied Biosystems #4367659) with forward and reverse primers (Table 2). Thermal conditions for qPCR were an initial 10-min activation step at 95°C and then 40 cycles of 15 s at 95°C denaturing and 60 s anneal/extend at 60°C. A dissociation curve (60 to 95°C) was performed to confirm the amplification of a single product. Quantification cycle (C_q_) thresholds were calculated using software (Agilent Aria v1.5) and normalised to a reference gene (*Gapdh*) using 2^-(ΔΔCq).

**Table 2:** Primers for RT-qPCR

Gene target	Forward primer (5′-3′)	Reverse primer (5′-3′)	Amplicon length (bp)	Transcript variants amplified	NCBI accession ID of first variant
**Human**					
*TUG1*	AGCGTGGGTGTACGTAAAGG	CCAAGGATTGGGGAACTGCT	82	1,2,3,6,7,8	NR_110492.1
*PPARGC1A*	TGAGAGGGCCAAGCAAAG	ATAAATCACACGGCGCTCTT	64	1-7	>NM_001330751.2
*TFAM*	GAACAACTACCCATATTTAAAGCTCA	GAATCAGGAAGTTCCCTCCA	95	1,2	>NM_003201.3
*GAPDH*	AGCCACATCGCTCAGACAC	GCCCAATACGACCAAATCC	66	1,3,4,7	>NM_002046.7
***Mouse***					
*Tug1*	GAGACACGACTCACCAAGCA	GAAGGTCATTGGCAGGTCCA	165	a,b,c	>NR_002321
*Ppargc1a*	TCCTCTTCAAGATCCTGTTAC	CACATACAAGGGAGAATTGC	78	1-9	>NM_008904.2
*Nrf1*	AAACAAAGGGTTTCATGGAC	GGTACGAGATGAGCTATACTG	149	1-10	>NM_001164226.1
*Gabpa (Nrf2)*	CAAGTATTGACAGTACCAGC	CTCTCTCTTCAATTTCTGCAC	127	1,4,5,9	>NM_207669.2
*Myod*	CTGGTTCTTCACGCCCAAA	CTGGAAGAACGGCTTCGAAGG	74	1	>NM_010866.2
*Myog*	GGGGCAATGCACTGGAGTTC	GCAGATTGTGGGCGTCTGTA	78	1	>NM_031189.2
*Gapdh*	GCCTGGAGAAACCTGCCAA	CGAAGGTGGAAGAGTGGGAG	144	1,3	>NM_001289726.1

### RNA sequencing and bioinformatics

RNA from two independent experiments each with three wells per LNA/EPS condition were used to generate RNA-sequencing libraries (1 well per library, *n* = 6). RNA first underwent clean-up (#R1013, Zymo Research) then concentration was confirmed fluorometrically (Qubit, Thermo Fisher) and RNA integrity number (≥9.5 for all samples) determined by electrophoresis (Tapestation, Agilent). Stranded libraries were prepared from 500 ng total RNA input with poly-A+ selection (E7760L, NEBNext® Ultra™ II Directional RNA Library Prep Kit for Illumina®) and included an SIRV Spike-In control, isoform E0 (#025.03, Lexogen) diluted in 1:500. Final library size and concentration were assessed (Qubit, Thermo Fisher and TapeStation, Agilent). Libraries were pre-sequenced (2 × 150 bp paired-end reads) on the MiniSeq Sequencer (Illumina, San Diego, CA) to obtain the read distribution. Each library was then re-pooled to equal molar concentrations, enzymatically treated, denatured and normalised to 2 nM. Finally, the normalised pooled library was sequenced with 150-bp paired-end reads on the Illumina NovaSeq 6000 platform (Deakin University Genomics Centre), generating at least 40 million reads per sample.

Reads first underwent adapter trimming and quality check with FastQC v0.11.7. Raw read quality filtering and adapter trimming was conducted with fastp v0.14.1 [55] (auto detect of adapters, trim_tail=1, poly_g_min_len=1, min phred quality 20, min length 36, u 90). Filtered reads were aligned to the mouse reference genome (*Mus musculus*, Ensembl version GRCm39.104) using STAR v2.5 [56] in 2-pass mode and expression quantified at the gene level and collated into a m × n matrix. Analysis of differential expression was performed on genes with ≥ 1 read per million (RPM) in at least 12 out of the 36 libraries using Voom/Limma in Degust v 4.1.5 [57]. Transcripts with a false discovery rate (FDR) < 0.05 were considered differentially expressed. Differentially expressed gene sets were assessed using PANTHER v16.0 [58] overrepresentation test (release 2021-02-24) with GO biological process annotations (GO Ontology database DOI: 10.5281/zenodo.5080993, release 2021-07-02) against the *Mus musculus* reference list with Fisher’s exact test with FDR correction. PGC-1α target genes were obtained from GTRD version 20.06 [59], based on ChIP-seq identification of genes containing one or more binding sites for PGC-1α (UniProt:Q9UBK2) in their promoter regions (TSS -1000, +100 bp). Transcription factor enrichment prediction analysis was performed using the ChIP-X Enrichment Analysis 3 (ChEA3) tool ranked by Fisher’s exact test (*p* value <0.05) with the ENCODE ChIP-seq library [60]. Gene overlap analyses were performed on lists of differentially expressed genes (FDR < 0.05) using the GeneOverlap package [61] in R (version 4.2.0).

### Western blot analysis

Protein was isolated from Tri-reagent lysates used for RNA analyses as described previously [62]. Briefly, 1-bromo-3-chloropropane (BCP) was added to an aliquot of TRIzol lysate (1:5 v/v) and shaken vigorously. Next, 2.3 volumes of 96% ethanol was added and vortexed, followed by additional BCP (1:6 v/v). Next, ddH_2_O was added (1:3 v/v), vortexed vigorously and centrifuged at 12,000 × *g* for 5 min at room temperature for phase separation. The upper aqueous layer was discarded, then ~ 2 volumes of 96% ethanol was added, vortexed vigorously and centrifuged at 12,000 × *g* for 5 min to pellet the protein. The supernatant was discarded, and the pellet allowed to dry at room temperature for 10 min. The pellet was then solubilised in 4% SDS buffer containing protease/phosphatase inhibitors (#P8340, Sigma Aldrich; #04906845001 PhosSTOP, Roche) with incubation at 50°C for 5 min to facilitate dissolution. Protein concentration was determined by BCA assay (#23225, Thermo Scientific). All samples were diluted to equal concentration (~1 μg/μL) mixed with reducing loading buffer (4x Laemmli sample buffer with 10% 2-mercaptoethanol). A pooled sample was made from all samples, then loaded into 5 separate wells (3–15 μg protein) to generate a standard curve on each gel (4–15%, Bio-Rad #5678085). Samples (~10 μg protein) and molecular weight marker (Bio-Rad #161-0373) were also loaded, then protein was separated by electrophoresis at 40 V for 30 min then 120 V for 60 min. The gel was imaged for stain-free total protein (Chemi Doc XR+, Bio-Rad) with ImageLab software (Image Lab v6, Bio-Rad) then proteins were transferred (Turbo Transfer system, Bio-Rad) to a methanol-saturated polyvinylidene difluoride (PVDF) membrane (Millipore Immobilon FL 0.45 μm #IPFL00010). The membrane was then PBS washed, dried for 1 h, re-saturated in methanol, washed with PBS, then blocked for 1 h (Li-Cor Intercept PBS blocking buffer). The membrane was then incubated with primary antibody in blocking buffer with 0.2% v/v Tween-20 overnight at 4°C. Primary antibodies and dilutions used were as follows: anti-Total OXPHOS Rodent (ab110413, Abcam, 1:1000), anti-pP38 MAPK Thr180/Tyr182 (9211, Cell Signaling Technology, 1:1000), anti-PGC-1α (ST1202, Calbiochem, 1:1000), anti-Myosin Skeletal-Slow/MyHC-i (M8421, Sigma, 1:5000), anti-Myosin 4/MyHC-iib (14-6503-82, eBioscience/Thermo, 1:1000), anti-citrate synthase (14309, Cell Signaling Technology, 1:1000), anti-OPA1 (NB1105529055, Novus Biologicals, 1:1000), and anti-Mitofusin-2 (9482, Cell Signaling Technology, 1:2000). Blots were then PBS-T washed incubated in anti-rabbit or anti-mouse IgG Dylight® 680 nm (5366S, 5470S) or 800 nm (5151S, 5257S) secondary antibodies (all from Cell Signaling Technology) at 1:10,000 in blocking buffer containing 0.2% Tween-20 and 0.01% SDS for 1 h at room temperature. Images were acquired (Odyssey® Infrared Imaging System, Licor, Lincoln, NE, USA) and blot densitometry performed using software (Odyssey v2.1, Licor). Blot density and stain-free total protein density for each sample was calculated by linear regression using the standard curve constructed from the pooled sample loaded on each gel. Calculated blot density values were then expressed relative to corresponding stain-free total protein density [63].

### High-resolution mitochondrial respiration analyses

C2C12 cells were grown to 90% confluence in 10-cm dishes then transfected with LNAs in proliferation or differentiation media for the indicated time. Myotubes were then harvested by trypsin-detachment, as described above, and resuspended in PBS. Cells were added to a high-resolution respirometer (Oxygraph O2k; Oroboros Instruments, Innsbruck, Austria) in each duplicate chamber at approximately 0.5 × 10^6^ cells/mL which contained MiR05 respiration media (0.5 mM EGTA, 10 mM KH_2_PO_4_, 3 mM MgCl_2_·6H_2_O, 60 mM lactobionic acid, 20 mM taurine, 20 mM HEPES, 110 mM D-sucrose and 1 mg/mL bovine serum albumin at pH 7.1) to a final volume of 2 mL that was maintained at 37°C with constant stirring. O_2_ gas was added to the chamber and media to maintain O_2_ between 350 and 250 μM. Cell membrane permeabilisation was achieved in-chamber using digitonin (4 μM) prior to commencing a substrate, uncoupler, inhibitor titration (SUIT) protocol similar to our previous work [15, 64]. Malate (0.5 mM) and glutamate (10 mM) were first titrated to assess O_2_ flux due to mitochondrial complex I leak (LEAK CI). Oxidative phosphorylation (state-3 respiration) supported by CI substrates (OXPHOS CI) was then determined with the addition of ADP (2.5 mM). Cytochrome c (10 μM) was added to confirm inner mitochondrial membrane integrity which was accepted as being ≤10% increase in O_2_ flux. This was followed by succinate (10 mM) to assess oxidative phosphorylation (state 3 respiration) supported by convergent complex I and II substrate input (OXPHOS CI+II). Uncoupled respiratory capacity of the electron transfer system supported by convergent complex I and II substrate input (ETS CI+II) was determined after titration of carbonyl cyanide p-trifloromethoxyphenylhydrazone (FCCP, 1.5–3 μM). Inhibitors of specific complexes were then applied: rotenone (0.5 μM) to inhibit CI resulting in ETS supported only by CII substrate flux (ETS CII), followed by the CIII inhibitor antimycin A (2.5 μM) to determine non-ETS O_2_ flux that was subtracted from values in all respiratory states. Oxygen flux values were normalised to total protein concentration of the cell suspension, as measured by BCA assay.

### Confocal microscopy

C2C12 cells were grown in 35-mm glass surface dishes (Fluorodish FD35, World Precision Instruments), differentiated for 3 days then transfected with *Tug1* or control LNA (25 nM) for 24 h. Mitochondria were labelled for 30 min with 50 nM MitoTracker RedCMXros (M7512, Life Technologies), then fixed with 2% paraformaldehyde in PBS for 10 min at 37°C. Fixed cells were then stained for F-actin with phalloidin (1:1000 in PBS, A22287 Life Technologies) and 4′,6-diamidino-2-phenylindole, dihydrochloride (DAPI) to visualise nuclei (1:1000 in PBS, #62248 Thermo Scientific). All control and *Tug1* LNA cells were prepared and imaged in parallel under the same conditions. Confocal microscopy (Nikon Eclipse Ti2, Australia) was performed using a 100x CFI Plan Apo Lambda NA 1.45 oil-immersion objective and Nikon DS-Qi2 camera. Excitation/emission wavelengths were 561/595 nm (MitoTracker), 640/700 nm (phalloidin) and 405/450 nm (DAPI), respectively. Z-stack images (0.15 μm steps) were acquired using software (Nikon Elements v5.21.03). Mitochondrial morphology in each Z stack image was quantified in ImageJ (NIH, Bethesda, MD) using a macro described previously [65]. Morphology parameters were calculated as follows: aspect ratio = major axis/minor axis, roundness = 4 × ((area)/(*π* × major axis^2^).

### Statistical analysis

All statistical analyses (except RNA-sequencing bioinformatics) were conducted using GraphPad Prism (v8.0; CA, USA). Data are presented as mean (SD) and analysed by two-way ANOVA with adjustment for multiple comparisons as described in the figure legend.

## Supplementary Information

**Additional file 1: Figure S1**: Expression of lncRNA *TUG1* transcript variants in male and female human skeletal muscle. **Figure S2**: Optimisation of *Tug1* knockdown in C2C12 myocytes and uncropped western blot images. **Figure S3**: Mitochondrial morphology in myotubes with *Tug1* knockdown, supplement to Fig. 3. **Figure S4**: *Tug1* knockdown in cells used for mitochondrial respiration experiments, related to Fig. 4. **Figure S5**: Supporting data for transcriptomic responses to EPS and *Tug1* KD, related to Figs. 6 and 7. Individual data are available at DOI: 10.6084/m9.figshare.20175770 [24].

**Additional file 2: Table S1**: List of differentially expressed genes with *Tug1* knockdown in C2C12 myotubes, *n*=6 per group. **Table S2**: Overrepresentation analysis of 867 differentially expressed genes due to *Tug1* knockdown in C2C12 myotubes. **Table S3**: List of differentially expressed genes (FDR<0.01, log2FC>1) at EPS 0h vs. Rest (in Control myotubes). **Table S4**: List of differentially expressed genes (FDR<0.01, log2FC>1) at EPS 3h vs. Rest (in Control myotubes). **Table S5**: List of genes upregulated at EPS 0h (FDR<0.05, log2FC>0.322) blunted by *Tug1* knockdown (FDR<0.05). **Table S6**: List of genes upregulated at EPS 3h (FDR<0.05, log2FC>0.322) blunted by *Tug1* knockdown (FDR<0.05). **Table S7**: Overrepresentation analysis of 53 genes upregulated at EPS 0h (FDR<0.05, log2FC>0.322) blunted by *Tug1* knockdown (FDR<0.05). **Table S8**: List of genes upregulated at EPS 0h (FDR<0.05, log2FC>0.322) augmented by *Tug1* knockdown (FDR<0.05). **Table S9**: List of genes upregulated at EPS 3h (FDR<0.05, log2FC>0.322) augmented by *Tug1* knockdown (FDR<0.05).

## Data Availability

All data generated or analysed during this study are included in this published article, its supplementary information files and publicly available repositories. RNA-sequencing data from this study have been deposited at the NCBI Gene Expression Omnibus under the accession number GSE185530 [66]. All other datasets generated in this study are available from the Figshare repository with the DOI of 10.6084/m9.figshare.20175770 [24].

## Abbreviations

AICAR: 5-Aminoimidazole-4-carboxamide ribonucleotide
*Alas1*: Aminolevulinic acid synthase 1
AMPK: 5′ Adenosine monophosphate-activated protein kinase
*Atf3*: Activating transcription factor 3
*Atp2a1*: Sarcoplasmic/endoplasmic reticulum calcium ATPase 1
ATP5F1A: ATP synthase subunit alpha, mitochondrial
CaMK: Ca2+/calmodulin-dependent protein kinase
ChREBP: Carbohydrate-responsive element-binding protein
*Cox7a1*: Cytochrome c oxidase subunit 7A1, mitochondrial
CREB: cAMP-responsive element-binding protein
*Dmd*: Dystrophin
EPS: Electrical pulse stimulation
*Esrrb*: Oestrogen-related receptor beta
*Esrrg*: Oestrogen-related receptor gamma
ETS: Electron transfer system, uncoupled
*Gabpa*: GA-binding protein alpha
*Hspa1a*: Heat shock 70 kDa protein 1A
*Kat2a*: Histone acetyltransferase KAT2A
LEAK: Leak state respiration
LNA: Locked nucleic acid
lncRNA: Long non-coding RNA
*Maff*: MAF bZIP transcription factor F
*Mcu*: Mitochondrial calcium uniporter
*Mcur*: Mitochondrial calcium uniporter regulator
MEF2: Myocyte-specific enhancer factor
MFN2: Mitofusin 2
*Mgst2*: Microsomal glutathione S-transferase 2
*Mic**u*: Mitochondrial calcium uptake
*Myf6*: Myogenic factor 6
MyHC: Myosin heavy chain
*Myod1*: Myogenic differentiation 1
*Myog*: Myogenin
NFAT: Nuclear factor of activated T-cells
*Nr4a1*: Nuclear receptor subfamily 4, group A, member 1
*Nr4a3*: Nuclear receptor subfamily 4, group A, member 3
*Nrf1*: Nuclear respiratory factor 1
OPA1: Optic atrophy protein 1
OXPHOS: Oxidative phosphorylation
p38-MAPK: p38 mitogen-activated protein kinase
P53: Cellular tumour antigen p53
PGC-1α: Peroxisome proliferator-activated receptor coactivator 1-alpha (protein)
*Polrmt*: Mitochondrial RNA polymerase
*Pparg*: Peroxisome proliferator-activated receptor gamma
*PPARGC1A*: Peroxisome proliferator-activated receptor coactivator 1-alpha (gene)
*Ppargc1b*: Peroxisome proliferator-activated receptor coactivator 1-beta (gene)
*Ppp1r3c*: Protein phosphatase 1 regulatory subunit 3C
RCR P/L: Respiratory control ratio, phosphorylating/leak
TFAM: Mitochondrial transcription factor A
TFB2M: Mitochondrial transcription factor B2
*TUG1*: Taurine-upregulated gene 1
UQCRC2: Cytochrome b-c1 complex subunit 2, mitochondrial
*Vgll4*: Transcription cofactor vestigial-like protein 4
*Vps13c*: Vacuolar protein sorting-associated protein 13C
