# Supplementary material for: Long non-coding RNA Tug1 modulates mitochondrial and myogenic responses to exercise in skeletal muscle
Source: BMC Biol. 2022 Jul 18;20:164. doi: 10.1186/s12915-022-01366-4 (PMC9295458; doi:10.1186/s12915-022-01366-4)
Supplement: Supplementary file 1 — Additional file 1: Figure S1: Expression of lncRNA TUG1 transcript variants in male and female human skeletal muscle. Figure S2: Optimisation of Tug1 knockdown in C2C12 myocytes and uncropped western blot images. Figure S3: Mitochondrial morphology in myotubes with Tug1 knockdown, supplement to Fig. 3. Figure S4: Tug1 knockdown in cells used for mitochondrial respiration experiments, related to Fig. 4. Figure S5: Supporting data for transcriptomic responses to EPS and Tug1 KD, related to Figs. 6 and 7. Individual data are available at DOI: 10.6084/m9.figshare.20175770 [24]. [file 12915_2022_1366_MOESM1_ESM.pdf]

A

Target	Primer (5'-3')	
hsa_TUG1_v1	Fwd	AGACAACGACTGAGCAAGCA
	Rev	TACCAGGTCTGTAGGTGAGTGG
hsa_TUG1_v2,3	Fwd	TGAATTATGTCCTGTGCCTCCT
	Rev	CCAGGTCTGTAGGCTGATGG
hsa_TUG1_v4,5	Fwd	GGAGGAGCCATCTTGTCTCG
	Rev	CAGGAGGCACAGGATGAGTG
hsa_TUG1_v6,7	Fwd	CGACTGAGCAAGCACTACCA
	Rev	ACCAGGTCTGTAGGCATAATTCA
hsa_TUG1_v8	Fwd	TGAGCAAGCACTACCACCAG
	Rev	CAGGAGGCACAGGATGAGTG

B

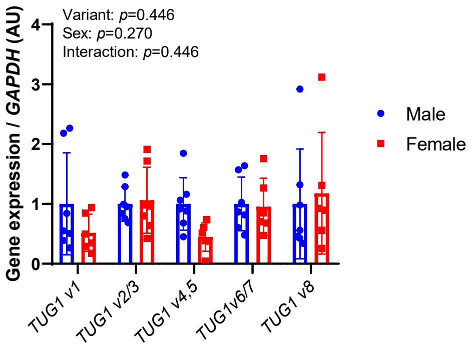

Figure S1: Expression of lncRNA *TUG1* transcript variants in male and female human skeletal muscle. A) Primers for individual or a combination of *TUG1* variant(s) were used to measure B) relative expression levels by RT-qPCR at baseline (pre-exercise). Bars are mean(SD) for n=7 subjects/sex, analysed by two-way ANOVA for main effects of sex and *TUG1* transcript variant. Individual data are available at DOI: 10.6084/m9.figshare.20175770.

A

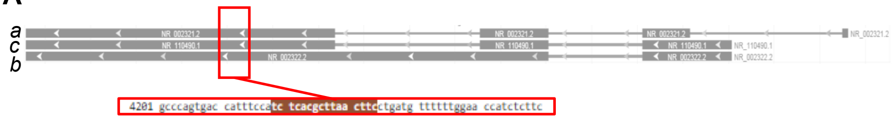

B

Target	Forward primer (5'-3')	Reverse primer (5'-3')
mmu_*Tug1*_a/b/c	GAGACACGACTCACCAAGCA	GAAGGTCATTGGCAGGTCCA
mmu_*Tug1*_a/c	CATAGTATCATCTTCGGGTTAC	CACAAAATGCATGTAGGTTC
mmu_*Tug1*_a	TCTTCTCGGCCAGAGACAAATGG	CAGAGGAAAGCCTTTTAACACCAC
mmu_*Tug1*_b	CATCTCACAAGGCTTCAACCAG	CCACTGTGTCACCTAGGAATTG

C

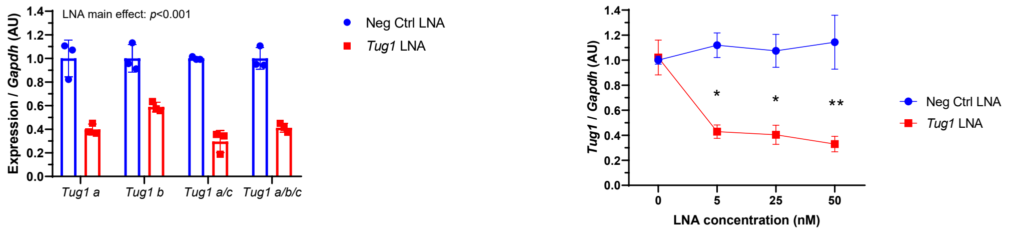

D

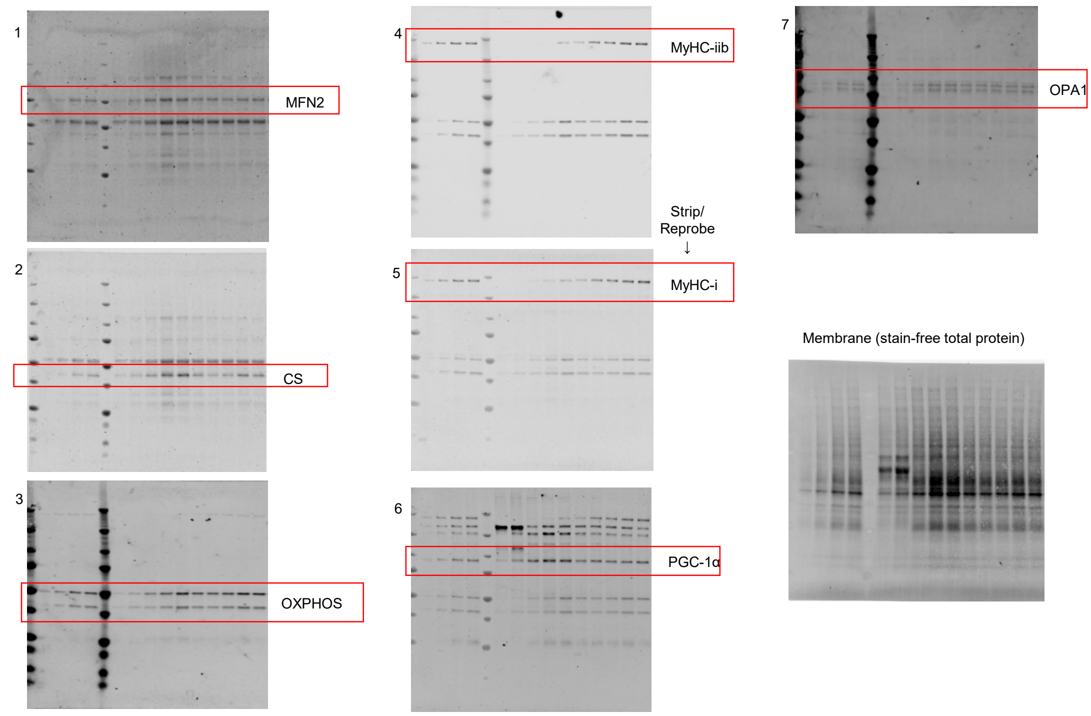

Figure S2: Optimisation of *Tug1* knockdown in C2C12 myocytes and uncropped western blot images. **A)** *Tug1* transcript variants *a*, *b* and *c* in *Mus musculus*. Red box denotes region that the *Tug1* antisense locked nucleic acid (LNA) binds. **B)** Cells were transfected with LNAs (25 *n*M) and differentiated for 24 h, then expression of *Tug1* transcript variants normalised to *Gapdh* was determined by RT-qPCR. **C)** Cells were transfected with various concentrations of *Tug1* or negative control antisense LNA GapmeRs for 24 h. *Tug1 a/b/c* expression was determined by RT-qPCR normalised to a reference gene (*Gapdh*). Data in B and C are mean(SD) for n=3 technical replicates, analysed by two-way ANOVA for main effect of LNA and variant (B) or concentration (C), significant interactions analysed with Bonferroni *post hoc* test: *p<0.05, **p<0.01, ***p<0.001 *Tug1* LNA vs. Neg Ctrl LNA. Individual data are available at DOI: 10.6084/m9.figshare.20175770. **D)** uncropped western blot images related to Figure 2 (C2C12 differentiation time course +/- *Tug1* KD). The number at top left of each image indicates the order in which the membrane was probed for each protein (leftover signal therefore appears in subsequent images). If antibody host species was different to the previous, signal was obtained with a secondary antibody of a different fluorophore wavelength (either 680 or 800 nm). Molecular weight markers (ladder) are 250, 150, 100, 75, 50, 37, 25, 20, and 15 kDa (top to bottom). Lanes 1 – 4 are a standard curve of a pooled sample. Lanes to the right of the second ladder are samples for increasing number of days of myotube differentiation time, from left to right: 0 (proliferation), 1, 2, 3, and 4; odd lanes are control samples and even lanes are *Tug1* KD.

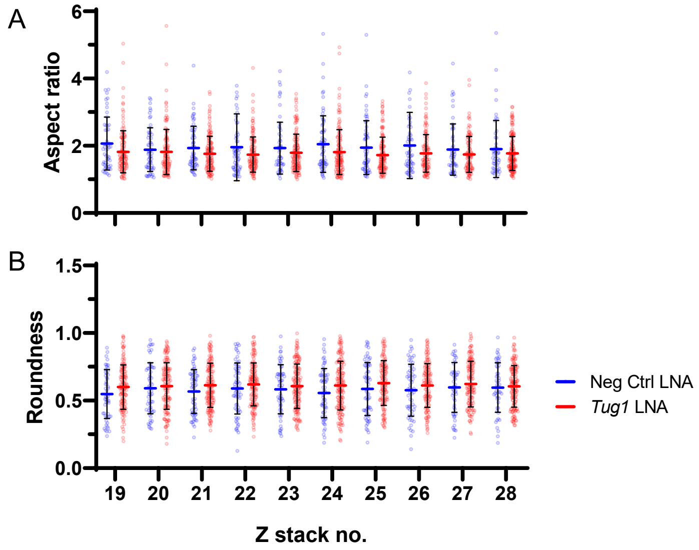

Figure S3: Mitochondrial morphology in myotubes with *Tug1* knockdown, supplement to Figure 3. Quantification of 10 consecutive central layers of a Z stack for A) aspect ratio (two way ANOVA main effect of LNA: $p<0.0001$), and B) roundness (two way ANOVA main effect of LNA: $p<0.0001$). Individual points represent individual mitochondrion regions, bars represent mean(SD) for one replicate. Individual data are available at DOI: 10.6084/m9.figshare.20175770.

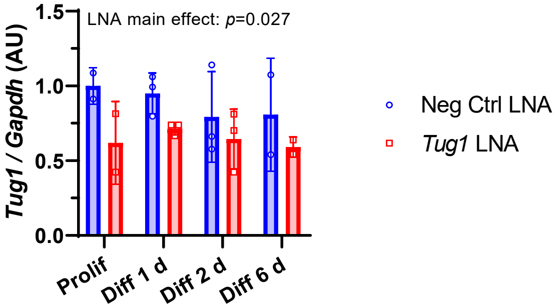

Figure S4. ***Tug1*** **knockdown in cells used for mitochondrial respiration experiments, related to Figure 4.** LNA transfection (10 nM) was performed 24 h prior to proliferation (Prolif) experiments, or at the beginning of the indicated number of days of differentiation (Diff). An aliquot of cells used for mitochondrial respiration experiments were lysed in Tri-reagent and stored at -20°C (note in one *Prolif* and one *Diff 6 d* experiment, an aliquot cells were not collected in Tri-reagent). RNA (500 ng) was reverse transcribed and analysed by qPCR as per *Methods*. Data are mean(SD) for n=2-3, analysed by two-way ANOVA. Individual data are available at DOI: 10.6084/m9.figshare.20175770.

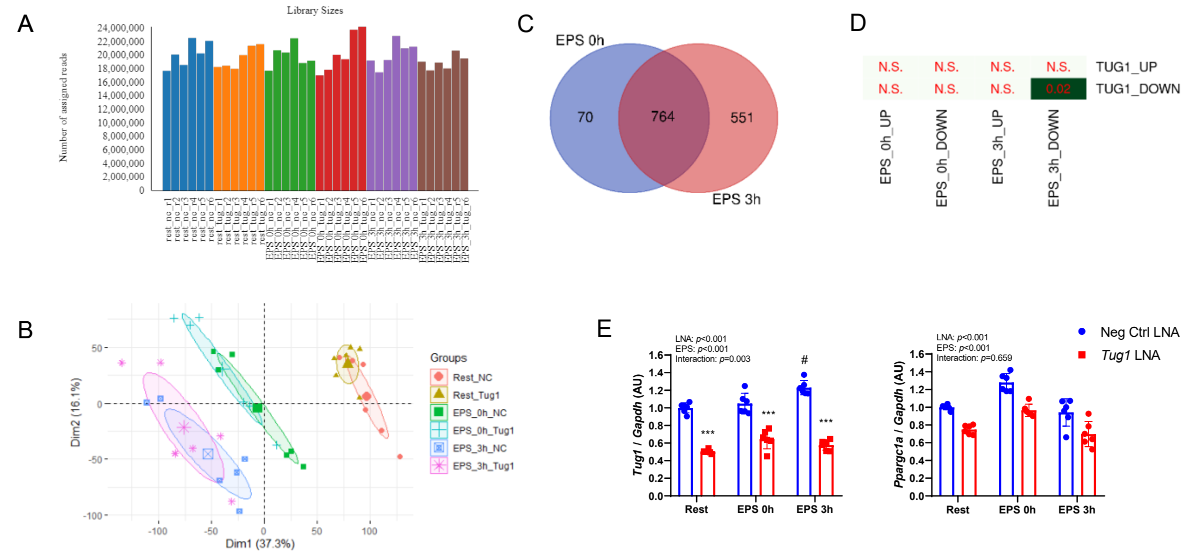

F

Gene	Bases aligned with LNA	Alignment with LNA	E value	Accession
Mus musculus taurine upregulated gene 1 (Tug1), transcript variant a, non-coding RNA	16/16	100%	2.2	NR_002321.3
Mus musculus T-box brain transcription factor 1 (Tbr1), mRNA	13/16	81%	139	NM_009322.3
PREDICTED: Mus musculus Casitas B-lineage lymphoma (Cbl), transcript variant X1, mRNA	13/16	81%	139	XM_006509963.4
PREDICTED: Mus musculus aryl-hydrocarbon receptor repressor (Ahrr), transcript variant X1, mRNA	12/16	75%	317	XM_006517048.3

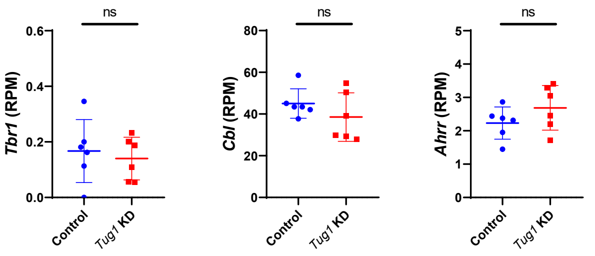

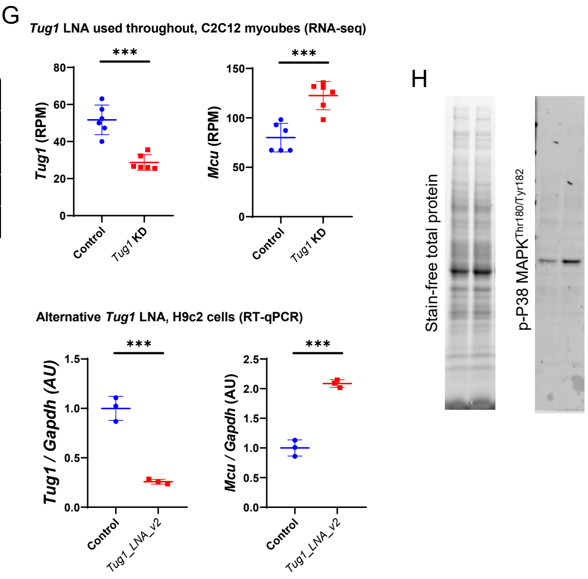

Figure S5: Supporting data for transcriptomic responses to EPS and *Tug1* KD, related to Figures 6 and 7.
A) Number of assigned reads for RNA-seq libraries, *n*=6 replicates per treatment and time point. **B)** Principle component analysis of RNA-seq libraries**. C)** Venn diagram showing the number of DE genes (log_2FC>1, FDR<0.01) in response to EPS at 0h and 3h relative to Rest. **D)** Gene overlap analysis of up or downregulated genes (FDR <0.05) between conditions; adjusted *p* value shown in red; N.S., not significant. **E)** Expression of *Tug1* and *Ppargc1a* measured by RT-qPCR; data are mean(SD) for *n*=6, analysed by two-way ANOVA for main effects of LNA and EPS, with Bonferroni *post hoc* test for significant interaction; ***p<0.001 *Tug1* LNA vs. negative control LNA; #p<0.05 vs. rest. **F)** BLAST search (top) for potential off-target genes (i.e. partial alignment with the *Tug1* LNA oligo sequence) and RNA-seq data (*n*=6/group) for these genes (bottom) which were not affected by the *Tug1* LNA mediated knockdown. **G)** Upregulation of *Mcu* observed with an alternative *Tug1* LNA oligo in a different cell line (rat H9c2), data are *n*=3 replicates per LNA, *Mcu* primers used: Fwd (5'-3') TTGTGCCCTCTGATGACGTG, Rev (5'-3') ACGGAGTCCGAGATAGGCTT, sequence for alternative LNA "*Tug1_LNA_v2*": ATTCAGTAGACAGCTA. **H)** Uncropped western blot image related to Figure 7. Individual data are available at DOI: 10.6084/m9.figshare.20175770.
